# Automatic Verification and Execution of Cyber Attack on IoT Devices

**DOI:** 10.3390/s23020733

**Published:** 2023-01-09

**Authors:** Fartein Lemjan Færøy, Muhammad Mudassar Yamin, Ankur Shukla, Basel Katt

**Affiliations:** 1 Department of Information Security and Communication Technology, Norwegian Univeristy of Science and Technology, 2815 Gjøvik, Norway; 2 Institute for Energy Technology, Os alle 5, 1777 Halden, Norway

**Keywords:** cybersecurity, IoT, penetration testing

## Abstract

Internet of Things (IoT) devices are becoming a part of our daily life; from health monitors to critical infrastructure, they are used everywhere. This makes them ideal targets for malicious actors to exploit for nefarious purposes. Recent attacks like the Mirai botnet are just examples in which default credentials were used to exploit thousands of devices. This raises major concerns about IoT device security. In this work, we aimed to investigate security of IoT devices through performing automatic penetration test on IoT devices. A penetration test is a way of detecting security problems, but manually testing billions of IoT devices is infeasible. This work has therefore examined autonomous penetration testing on IoT devices. In recent studies, automated attack execution models were developed for modeling automated attacks in cyber ranges. We have (1) investigated how such models can be applied for performing autonomous IoT penetration testing. Furthermore, we have (2) investigated if some well known and severe Wi-Fi related vulnerabilities still exist in IoT devices. Through a case study, we have shown that the such models can be used to model and design autonomous penetration testing agents for IoT devices. In addition, we have demonstrated that well-known vulnerabilities are present in deployed and currently sold products used in IoT devices, and that they can be both autonomously revealed through our developed system.

## 1. Introduction

Penetration testing describes the attempt of hacking a computer system in order to uncover potential vulnerabilities and thus assess its level of security. Because a penetration test is mostly done manually, it is costly and time-consuming, and the results will depend on the incline and expertise of the tester. A fully automatic penetration test would be fast, inexpensive, and deterministic. In addition, it would be highly useful for training purposes in a cyber range [1]. However, existing research has proven autonomous penetration testing to be a complex task and especially challenging for devices in the Internet of Things (IoT) domain.

Researchers [2] have developed a new modeling plan to subdivide the task of autonomously attacking and defending systems in a cyber range network. In the study, they demonstrated how agents on both sides could employ the modeling plan with a selected number of offensive and defensive tactics. This research aims to expand and investigate the capabilities of autonomous attack agents created in the work of Yamin & Katt [2] by both introducing new types of attacks and using the agents to automate penetration testing on IoT devices. This work will try to expand the capabilities and usability of the attacking agent. This will be done by employing the modeling plan on an exploit, targeting a vulnerability in an IoT device. Finally, the new attack agent is tested on running IoT devices and is compared with current automated vulnerability scanners and exploitation systems.

In this paper, we will first share the background and related work to the IoT threat landscape. Continuing that, we will provide details of the case study environment in which a manual cyber attack on IoT devices is performed and share its results. Moving forward, we will present the system design for automated attack and verification on IoT devices. We will then discuss the results and conclude the article.

## 2. Background and Related Work

### 2.1. Internet of Things and Cybersecurity

One may think that the security breach of a smart light bulb would be inconsequential, but there are numerous risks associated with the successful attack on IoT devices. In this Section, we will address the importance of IoT security and why it is difficult, along with potential attack vectors, threat actors, and existing counter measures.

#### 2.1.1. Threats and Risks

If an IoT device is compromised, then perhaps most evident issues come from the potential loss of availability as defined in the CIA Triad [3]. As mentioned, IoT devices are used for an extensive number of applications. In the case of smart homes, a breached device can imply a broken refrigerator, unknown access to home security cameras, or an open smart lock on a front door or a safe. A broken sensor or actuator in a factory can halt production or endanger workers and end-users. In terms of the IoMT (Internet of Medical Things), the negative implications on availability in equipment like modern pacemakers and insulin pumps can have fatal consequences [4].

Many IoT devices handle sensitive information. When used in commerce, this could be customer information, physical security information, or industry secrets that could be valuable to criminals or competitors. Devices sold to consumers may handle and store sensitive information about the owner. For instance, data processed by a smart thermostat may reveal when a house is vacant [5], workout devices may handle sensitive health data about the owner, and home surveillance cameras may record continually, even when the owners are home. Stolen data from such devices may be used in profiling for criminal purposes, identity theft, blackmailing, spear-phishing, or weakening the security of a physical place of residence or business. A security researcher also revealed that a smart light bulb stored network access keys in plaintext, which were retrievable even after the light bulb was disconnected [6]. Similarly, a compromised device can act as an entry point into a system or network [5].

IoT devices have also been the main target for different types of malware. In 2016, the largest DDOS attack ever recorded at the time was delivered by a botnet named Mirai [7]. It consisted of over 300,000 infected IoT devices, and the attack brought down several global services including GitHub, PayPal, Amazon.com, BBC, PlayStation Network, and Spotify. Following the attack, the source code of the botnet malware was published on Hack Forums and GitHub, allowing anyone to recreate the Mirai malware or use parts of it in their own malware [8,9]. IoT devices are continually operative and connected to the Internet while often requiring minimal human interaction; they are highly favorable for botnets and malware [10]. Botnet devices can be used for DDOS attacks, cryptomining, password hash cracking, or other types of distributed computing. While it is unlikely that the owner is persecuted for the malicious acts of an infected device, the device itself may become slow, malfunction, or take damage from overheating. In addition, the botnet administrator may extract sensitive information or in other ways misuse the device.

#### 2.1.2. Threat Actors

As IoT devices are used in many different fields, from smart homes to billion-dollar industries and government projects, the potential threat actors are similarly diverse. Background, funding, motivation, skills, target, and scope will vary between different types, which we can categorize under groups described below [11]. They are loosely sorted in ascending order based on their cybersecurity threat level for companies, governments, and other high-value targets. The last two groups are the most likely to act as an APT (Advanced Persistent Threat) once compromising a device, posing as a major threat unbeknownst to the victim over weeks, months, or even years [12].

1. **Cyber-criminals** is a term used for individuals that conduct illegal activities on the Web that does not involve hacking. This includes drug dealing, human trafficking, sharing or downloading child pornography, and conducting financial fraud. While they are not directly a cybersecurity threat, they are criminals within the cyber realm and are included in this list for the sake of completeness.
2. **Script kiddies** and **cyber-punks** have limited knowledge and skills and use existing tools to exploit low hanging fruit. Fame among peers, small gains, or simply entertainment are usually their motives.
3. **Hacktivists** are the digital equivalent to activists. They consist of anonymous groups that target private organizations and governments to publicize a political agenda.
4. **Cyber-terrorists** are terrorists using the web to recruit new members and share information. They may also conduct attacks in the cyber domain with the same motives as terrorist attacks in the physical world.
5. **Black hat hackers** are mostly individual hackers with knowledge and expertise in hacking and the tools used. Their targets may be specific companies or individuals or arbitrary devices found by means such as the search engine Shodan. Their motives are usually reputation or financial gains.
6. **Malware- and hacking tools coders** are highly skilled adversaries that create tools and malware used to target different types of systems. They may work alone or in a criminal organization. The may sell the tools or use them in ransomware attacks or to create botnets. This is one of the most prevalent threats for IoT devices [13].
7. **State-sponsored attackers** are groups with extensive expertise and resources. They target corporations or governments in order to reveal trade or state secrets, plans, or ideas, or in other ways harm the victim. Their attacks are sophisticated and may utilize zero-days, making them difficult to avert.

Knowing where the threats come from is important in order to understand how the security of a system should be adequately tested. The motivation and skill of an adversary will determine its targets and attack vectors. Due to the diverse set of threat actors, it is important that security audits test the breadth of potential attacks as they may come from any of these adversaries.

#### 2.1.3. Attack Surface and Security Issues

To map the attack surface of a system, one should consider the various parts of the system, and how it operates. Due to the numerous applications and heterogeneous environments of IOT, researchers, and developers have not been able to universally agree on a single architecture for describing IoT devices [14]. The approach described in this work is a three-layer model that differentiates between the perception, network, and application layer [15,16]. The attack vectors used on each of the three layers are highly different from each other, due to which the model is especially useful when investigating topics related to penetration testing [16].

- Perception Layer
  The physical part of the device is represented by the perception layer. The layer gathers information from and interacts with the physical world around it. To achieve this, the device can use sensors, actuators, GPS, RFIDs, or other similar technologies. IoT devices may often have limited computing powers, storage, and battery capacity. This limits the complexity of its encryption schemes and key lengths [17]. Furthermore, the devices may need to be small and lightweight which limits the possible physical hardening options. The IoT devices may be situated in places where maintenance is difficult or neglected because they rarely receive human interaction. Such devices may be left untouched simply because they work, contributing to the growing concern of orphaned devices [18].
- Network Layer
  To control the actuators or process the information gathered in the perception layer, data must be transmitted between the physical and the application layer. The network layer connects the end nodes to network devices, servers or other IoT objects, and handles the corresponding data flow. The connection is often wireless due of cost, coverage, and mobility. Examples of wireless technologies used within this layer include ZigBee, Bluetooth, WiFi, 4G, 5G, satellites, and combinations of them. The network layer is prone to jamming attacks, access point spoofing, data sniffing, MITM (Man-in-the-Middle) attacks, and more [17]. The devices may be used as an entry point into their connected networks, which makes their security increasingly important [16].
- Application Layer
  The application layer provides user interaction and management of the service provided by the IoT device. This may be presented as a smart home hub, a Web or mobile application, or a machine-to-machine interface. Depending on the field, service, and user, there are numerous technologies and applications that can be used on this layer. Because the application layer often presents an interface to the Internet, it has the same security issues as most other computing devices with an Internet connection. While this layer can be harder to exploit, it will often be accessible from anywhere in the world, substantially increasing the potential threat actors. Examples of common attacks include credential guessing, SQL injection, buffer overflow, and social engineering attacks [19].

### 2.2. Penetration Testing

A penetration test is a form of security audit that can be performed to ensure an appropriate level of security of a system [20,21]. It is a form of stress test that usually attempts to bypass or break the authentication mechanism of the device, or in other ways compromise its integrity, availability, or confidentiality. This is accomplished by discovering vulnerabilities in hardware, software, or communication protocols. A penetration tester may exploit the discovered vulnerabilities as a proof of concept or elevate its privileges within the system to reveal more vulnerabilities. The test can be defined as either *black box* or *white box* [20]. The former suggests the tester knows nothing about the underlying systems and acts as an external attacker. In a white box penetration test, the attacker has some kind of inside knowledge or access, like a software source code. In this work, we will only consider black box penetration testing because this method emulates hacking attempts as they are realistically performed. Penetration tests are commonly performed by one or more experienced penetration testers who employ a broad set of hardware and software tools, depending on the target system [21]. The software tools are used to scan the target in order to both map the system and discover potential vulnerabilities. Some tools are capable of exploiting vulnerabilities. Essentially, the software tools are automating parts of the process, but they require interaction to both run and interpret the output. A penetration test on a system will have a large number of potential attack vectors and surfaces to test. The action space of possible steps to scan and exploit is vast, while the outcomes may have severe implications for the system and people compromised by the test. Therefore, a structured procedure should be followed to ensure the outcomes are correct and the interests of all afflicted parties are accounted for. To standardize the procedure, several methodologies have been suggested [22]. A commonly acknowledged and utilized methodology is the PTES (Penetration Testing Execution Standard) [23]. This standard describes the penetration test in seven steps:

1. **Pre-Engagement Interactions**
  This step involves and emphasises the importance of clearly defining the target, scope, and potential boundaries before interacting with a system.
2. **Intelligence Gathering**
  Before attacking the system, the tester must know how it functions, how it is structured, and how it can be interacted with. As mentioned above, automated software tools can often be used for this purpose.
3. **Threat Modelling**
  To properly analyze the security of a system, the tester should know who could attack it and why. Thus, PTES threat modelling focuses on the assets that can be targeted and the liable threat actors.
4. **Vulnerability Analysis**
  This step is where potential vulnerabilities, from misconfigurations to faulty designs, are uncovered. Many software tools, as well as human interaction with the system, are important to properly examine it.
5. **Exploitation**
  To analyze the discovered vulnerabilities, the tester will attempt to exploit them. This should reveal their potential implications and may be used to uncover more vulnerabilities.
6. **Postexploitation**
  If a component of the system has been successfully compromised, the value of the component should be evaluated with regards to its usefulness in further exploitation of the system.
7. **Reporting**
  The value of the penetration test comes from reporting the discovered and exploited vulnerabilities. These should be evaluated according to their severity and risk.

#### 2.2.1. Testing of IoT Devices

Penetration testing IoT devices is not much different from penetration testing of larger computer systems. The audit must inquire all three layers described in Section 2.1.3, which would be the case regardless of the target system. However, the tests performed on the network layer and particularly the perception layer may be different depending on the device tested. Due to the vast number of different applications and utilities provided by the IOT, there is a corresponding diversity in the various technologies utilized. The devices may have particular vulnerabilities related to their services, location, sensors, communication protocol, and so on.

The steps of the PTES discussed above can be applied to the penetration testing of IoT systems as well. The threat actors described in Section 2.1.2 are an important part of the threat modeling step, while the IoT architecture and security issues from Section 2.1.3 are essential when evaluating step 2, 3, and 5 of the PTES model. Other models and methodologies designed specifically for penetration testing within the domain of IoT have recently been proposed [16,24,25,26].

#### 2.2.2. Autonomous Penetration Testing

As discussed, penetration tests are traditionally performed manually, which makes them time-consuming and expensive with potentially unreliable results. Autonomous penetration tests would solve these issues and be beneficial for development, production, certification, education, and research. While autonomous tools exist, they are either used for a specific purpose within penetration testing, require some level of human expert interaction or are not as capable as a human penetration tester. The main reason for automated penetration testing still being an open challenge comes from the large action space in which such an agent would operate [27]. The task becomes even more difficult when accounting for IoT devices due to their different applications and heterogeneous environments. The current state of the art and related work will be discussed in more detail in Section 2.5.

### 2.3. Wi-Fi

Wireless Fidelity, commonly abbreviated to Wi-Fi, is a wireless communication technology based on the IEEE 802.11 standards for LAN [28]. Wi-Fi allows devices equipped with a wireless NIC to act as clients, connecting to a local AP over the air interface. The AP will often provide Internet access to its clients, but it may also simply create a local network in which the stations can communicate. The clients and APs in a LAN are called *stations*. To distinguish individual networks in a WLAN, the term SSID (Service Set Identifier) is often used [29]. This describes the network name which is usually how a Wi-Fi user will address and identify networks. In this work, we will refer to the term ESSID (Extended SSID) for a network name to clearly separate it from the BSSID (Basic SSID), which describes individual APs MAC addresses. ESSID technically describes the set of all BSSIDs in a network, but for the practical context of this work, the terms SSID and ESSID can be considered equivalent. The term ESSID is also employed by the case study software tools discussed in Section 3. Some IoT devices are connected to the Internet through a Wi-Fi connection. Other devices may run their own Wi-Fi network to provide Internet access, like wireless routers, mobile hotspots, or to transmit data to its clients.

#### 2.3.1. Encryption Standards and WPA2 Personal

There are several generations of encryption schemes that can be employed on a Wi-Fi connection. WPA version 3 is the most recent and secure [30]. Still, many APs run on WPA2, which has several vulnerabilities associated to it [31,32,33]. WPA2 is based on IEEE 802.11i and was ratified in 2004 [34,35]. It can be further differentiated between its modes *Personal* and *Enterprise*, where the latter has a few additional security features. Enterprise mode utilizes an authentication server that administers individual session keys and is designed for larger networks of upwards of 10 stations [36]. The WPA2 Personal is sometimes referred to as PSK mode because the AP authenticates the user and encrypts the connection based on a single key known by all stations in the network [31].

A 2022 study suggested that private WLANs are predominantly encrypted using WPA2 or less secure protocols, four years after the publication of WPA3 [37]. When probing 21,345 WLANs in Cyprus, they found that only 13 networks employed WPA3. 74.7% used WPA2 or WPA2/WPA in mixed mode meaning that devices will choose WPA2 if they support it. Only a 0.2% used WPA or the older WEP, while 25.1% had no encryption. Similar studies from 2019 show comparable results in Romania and Bulgaria where WPA2 was distinctly the most popular protocol [38,39]. While these results may be slightly different in other countries, they show that WPA2 is still common and that we can expect millions of devices to communicate over this encryption scheme for several years forward.

#### 2.3.2. The 4-Way Handshake

**Figure 1:**
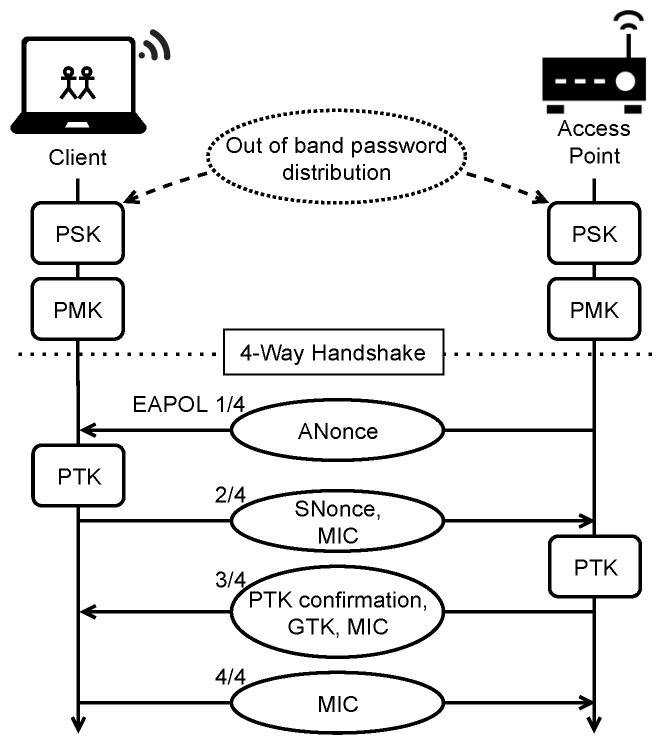
The 4-way handshake.

WPA2 Personal encryption is based on CCMP (Counter Mode with Cipher Block Chaining Message) with the AES block cipher [31,34]. Unicast messages are encrypted using a PTK while a GTK is used for multicast and broadcast messages on the network. The process of authenticating and negotiating these keys is known as the *4-way handshake*. Its name comes from the four EAPOL (Extensible Authentication Protocol over LAN) messages, which constitute the process, transmitted between the client and AP as seen in Figure 1. The roles are often labeled *supplicant* and *authenticator* during the handshake [34], but we will stick to the terms client and AP as they are used throughout the context of this project.

Upon finishing the initial connection process, the handshake authentication is begun. Both parties derive the PMK from the PSK, which is distributed out of band. The first message of the handshake contains a random 128-bit value called *ANonce*, which is sent from the AP to the client. The client generates its own random value called *SNonce*. The client now knows the two random values, the PMK and the BSSIDs which are the MAC addresses of the two stations. With this information, the client can calculate the PTK. The client then transmits the SNonce with a MIC created using the PTK. The AP can now also calculate the PTK and then utilize it to verify the MIC. If validated, a third EAPOL message is sent from the AP. This contains a confirmation of the PTK, a GTK encrypted with the PTK, along with a MIC. Finally, the client replies with a MIC, completing the 4-way handshake.

#### 2.3.3. Deauthentication Frames

A type of management frames called the *deauthentication* frame is used to instruct a station to drop its network connection [40]. Usually, the station will automatically attempt to reconnect, which initiates a new handshake process. However, if a client continuously receives deauthentication frames from the AP, it will not be able to establish a new connection. This constitutes a serious concern because in the IEEE 802.11i standard, which WPA2 is based on, the stations do not authenticate or encrypt management and control frames [35]. The frames are sent in plain-text, meaning that an adversary can forge a deauthentication frame that appears to be from an AP in order to kill the connection to a client [40]. The adversary can then capture the handshake process upon reconnection or continue to send the deauthentication frames. The latter would act as a DOS attack by withholding the client from reconnecting.

In 2009, the IEEE published the 802.11w version, which addressed the lacking protection of management frames [34]. With these changes, management frames should be protected if possible. To facilitate these security aspects, the third messages of the WPA handshake would contain an encrypted IGTK along with the GTK. The changes to the 802.11 version allows a station, i.e., a client, to drop the deauthentication frame if an IGTK check fails. Essentially, this means that an adversary that does not know the IGTK can not deauthenticate a station by simply spoofing the BSSID of the other connected station.

#### 2.3.4. Evil Twin Attack

If a client is disconnected from its AP, it will usually attempt to reconnect automatically. Originally, the client would only check if the ESSID of the network it disconnected from matches any networks within range and attempt to reconnect if it finds a match [41]. If there are more than one network with the same ESSID, it will connect to the one with the strongest signal. This is a severe vulnerability because the ESSID is visible to anyone within range, making it trivial to create a matching Wi-Fi network. Impersonating an AP to target unaware clients is called an *Evil Twin attack* and it can be classified under the serious category of MITM attacks.

**Figure 2:**
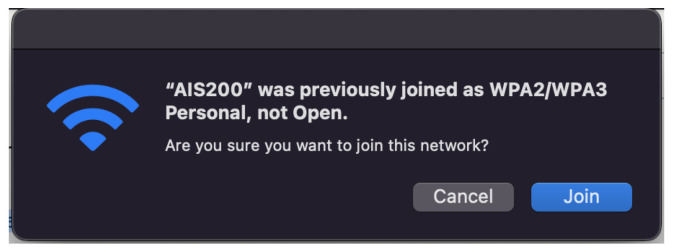
Warning issued when attempting connection to a rogue AP with no encryption scheme.

Because of severity of this attack, many new devices will now examine the encryption scheme of the AP. If the ESSID of the network matches a previously associated network, but the encryption scheme is different or removed, it will not connect automatically. Furthermore, if attempted to connect manually, the device will issue a warning as shown in Figure 2. Because of this, a successful Evil Twin attack on an encrypted network will usually require knowledge of the encryption key used.

### 2.4. The EP Model and Formal Specification

The Execution Plan model was developed by Yamin and Katt to specify and describe the decision making process of autonomous agents within a cyber range [42]. The model has a tree structure with three levels. The tree translates the high-level goals of the agent into concrete commands for the agent to perform. The results when running EP model are either *plan fulfilled*, *plan not fulfilled*, or *plan maybe impractical*.

**Level 1** contains the root node of the tree. This describes the main goal of the agent. Connected to the main goal and within the first level are one or more branches describing subgoals. These are separated by the logical operators ∧ (*and*) and ∨ (*or*), which determine whether one or all subgoals must be achieved in order for its parent goal to succeed.

**Level 2** describes *actions* and *conditions* that will eventually decide what commands will be executed. These conditions are Boolean, meaning they can only be answered with *yes*/*true* or *no*/*false*. As a consequence, the ∨ operator is the only allowed operator within this level. Each root node in the second level has its own subtree that corresponds to a subgoal in the upper level. Each of the second level leaf nodes are either a “Not fulfilled”-node or an actions-node. The former has no children and implies that the conditions to fulfill the plan have not been met, while the latter has one or more children in the next level.

**Level 3** contains a single layer of nodes which describes concrete commands. These commands execute the actions described by the parent nodes in the layer above. The siblings within the third layer are separated by either the ∧ operator, which implies that all commands must be executed for the plan to be fulfilled, or the ∨ operator, implying that either of them is sufficient. If a command is not executed successfully, the model indicates that the plan may be impractical.

#### 2.4.1. Formal Specification and Verification

Formal specification within computer science is used to describe system requirements and function through abstraction and mathematics [43]. This is useful to aid with the design and testing of the system before, during, or even after development. Formal specification can reduce redundant and ambiguous specifications and facilitate the development of more effective code with less errors [44]. While code verification like unit tests can demonstrate that code modules work as intended, the tests may not detect logical flaws in the system design. By abstracting the software and its elements, developers can verify that the proposed design works as intended before writing the code and its details. The EP model can and should be formally verified to ensure that its logic is correct and the final states can be reached. For the purpose of verifying the EP model within this work, the formal specification language called TLA+ (Temporal Logic of Actions) [45] will be used. TLA+ is a formal specification language developed by Leslie Lamport for use in the design and verification of distributed systems. It is a high-level language that allows users to describe the behavior of a system in a precise and unambiguous way. There are several reasons why TLA+ may be considered better than other formal specification methods:

1. TLA+ is expressive: It allows users to describe complex systems and their behaviors in a clear and concise way.
2. TLA+ is modular: It allows users to divide a system into smaller, easier-to-manage components, making it easier to understand and verify.
3. TLA+ is reusable: Because it is a high-level language, TLA+ specifications can be reused and adapted to different systems, saving time and effort.
4. TLA+ is supported by powerful tools: The TLA+ Toolbox is a suite of tools that support the development and verification of TLA+ specifications, including a model checker and an automatic theorem prover.

#### 2.4.2. TLA+

A formal model written in TLA+ is called a *specification* [45]. The specification is a mathematical interpretation and abstraction of the discrete events of a system, which, in this case, will be the decision making process of the agent. The fundamental elements of a specification are the *states*. A state describes the variables of a system at a specific point in time during system operation. The transition from one state to the next is often what would be described as a program event. In TLA+, this transition is called a *step*. The full system execution will be represented by a specific sequence of states where the first state is the *init* state. The init state represents the initial condition, and all successive steps must adhere to the rules described by the *next-state relation*. A state explosion [46] is a phenomenon that occurs when the number of possible states in a formal model becomes too large to be manageable. It can be a major challenge when it comes to analyzing and understanding complex systems. One way to tackle state explosions in formal models is to use abstraction techniques [47], which involve simplifying the model by ignoring certain details that are not essential to the problem at hand. This can help to reduce the number of states and make the model more tractable. Another approach is to use heuristics, which are rules of thumb or shortcuts that can help to guide the analysis of the model. Heuristics can be useful in identifying key features or trends in the data and can help to reduce the complexity of the model. Other methods for tackling state explosions in formal models include using automated tools, such as model checkers and theorem provers, to analyze and verify the model, and applying machine learning techniques to learn from data and make predictions about the model. We used the TLC Model Checker to evaluate all possible states that can be reached beginning in the init state. The state transition diagram displays all reachable states as nodes, while their sequence is described by directed edges between them.

### 2.5. Related Work

As mentioned in Section 2.2, a number of tools that automate parts or most of the penetration testing already exists. Examples of partial automation tools include Nmap, which can be used to scan open ports and identify services on a given system [48], and SQLmap, which can test for SQL injection vulnerabilities and more [49]. Proprietary tools like Metasploit Pro [50] and Nessus [51] have automated vulnerability scanning and exploitation capabilities. However, these tools require some level of interaction with a human penetration tester. Numerous attempts have been made at fully automating penetration testing. In the current state of research, many are attempting to solve the issue by employing AI and specifically machine learning techniques. The first hurdle to overcome when developing such solutions is the vast action space in which the agents must train and operate. In 2021, a cybersecurity researcher and former penetration tester developed and trained an autonomous penetration testing agent using deep RL algorithms [52]. Modules within a penetration testing software called Metasploit were used to create an abstraction of the action space. Within a testbed containing a highly vulnerable machine, the agent was able to acquire root privileges in all test runs. However, a control agent using the same modules were able to reach this access level in more than 20% of the test runs when randomly selecting attack vectors.

Similar work has been done by Schwartz and Kurniawati [53], Zennaro and Erdod [54] and Hu et al. [55], which involved various Q-learning algorithms to train RL penetration testing agents. Another suggestion proposed by Tran et al. investigates an algebraic approach to structure the action space hierarchically [56]. Within this abstraction, they trained the penetration testing agent using deep RL algorithms. Their experiments showed positive results when compared to the deep Q-learning approach. While the machine learning methods have shown positive results, even in large action spaces, the environments in which they have been trained and tested are still limited in scope of all computing systems. Especially, when accounting for all the various IoT devices and their applications. Moreover, new services and vulnerabilities are discovered every day. Limited research has been done on how these agents adapt and perform in volatile environments.

Another issue comes from the risk of damaging systems as penetration tests are often performed on live services and systems. A human penetration tester would both consider the implications of an exploit, and only execute it on a single device as a proof of concept if it is considered safe to perform. An autonomous agent will have less understanding of potential consequences. When a RL agent learns that exploiting a certain vulnerability provides a reward, it will attempt to perform this exploitation whenever possible. Some work has been done related to the automated penetration testing of IoT devices specifically. Chu and Lisitsa proposed the use of a BDI model to map the goals and plans of a penetration test to concrete actions and perceptions of the target system [16]. An autonomous agent will then make the decisions based on an AI framework called *procedural reasoning system*. Rak et al. developed an automatic threat modeling system which would describe concrete actions to manually perform the penetration test [26]. The actions and instructions were intended to be easy enough for a smart home owner without training to test the vulnerabilities. Considering IoT devices often are part of a larger network, Yadav et al. developed a penetration testing framework for analyzing both the IoT devices and the their connected network in its entirety [27].

Some researchers used Attack trees [57], which are a graphical tool used to model the ways in which a system can be attacked. They are commonly used in the field of cybersecurity to identify and assess potential threats and vulnerabilities. They help to identify the assets that need to be protected, such as sensitive data or critical infrastructure. Determine the potential attacks that could compromise these assets. These attacks can be grouped into categories, such as physical attacks, network attacks, or software vulnerabilities. For each attack category, identify the specific attacks that could be used to compromise the assets. These attacks can be represented as branches on the attack tree. For each attack, identify the prerequisites or subattacks that would be required to execute the attack. These prerequisites can be represented as additional branches on the attack tree. Continue adding branches to the attack tree until all possible attacks and prerequisites have been identified. Evaluate the likelihood and impact of each attack, and prioritize the ones that pose the greatest risk. Develop strategies to mitigate or eliminate the identified threats. This may include implementing security controls, strengthening security policies, or educating users about potential threats [58].

For securing IoT infrastructure, Blockchain technology can be used in a number of ways [59]. It can be used to verify the identity of IoT devices and ensure that only authorized devices are able to access the network. This can help to prevent unauthorized access and protect against attacks. Its decentralized and distributed nature makes it well-suited for storing and verifying data from IoT devices. It can help to ensure that data is not tampered with or altered in any way, making it more reliable and trustworthy. It can be used to manage the distribution of software updates to IoT devices in a secure and controlled manner. This can help to ensure that devices are always running the most up-to-date software and are not vulnerable to attacks [60]. It can be used to encrypt and transmit data between IoT devices in a secure manner, helping to protect against interception or tampering.

There have been several studies related to WLAN and IoT security. Hossain et al. presented and discussed various security issues and challenges in the IOT, including WLAN connectivity [17]. In 2020, Kristiyanto et al. analyzed a deauthentication attack on a Wi-Fi connected IoT camera [40] equivalent to the DOS attack presented in Section 5.1. Verma et al. demonstrated several serious security risks for IoT devices on connected to IEEE 802.11ah WLAN networks [61]. Vanhoef and Piessens demonstrated that an adversary can force a reinstallation of the key generated during the handshake process, effectively resetting the nonce and replay counters [62]. In WPA2 PSK-CCMP, this would allow the adversary to decrypt and replay frames but not forge new ones. In 2021, Vanhoef discovered more vulnerabilities in both design and common implementations of all WPA versions [63]. With user interaction, an adversary uses the design vulnerability to steal sensitive data. Furthermore, Vanhoef demonstrated how the vulnerabilities could be exploited to attack IoT devices. Over the last years, some tools that automate attacks on the Wi-Fi related vulnerabilities have been developed. Specifically, we know of the “WiFi Exploitation Framework-WEF” [64], “Airgeddon” [65], and “Wifiphisher” [66]. These are all capable of running several attacks on WPA2 including versions of the Evil Twin attack. Yet, to the best of our knowledge, we do not think they are able to fully automate the process of the second attack presented in Section 5.1.

## 3. Case Study and Environment

A laptop with the Kali Linux operating system was used as a basis of the attacks. Kali Linux is an open-source Debian based distribution of Linux, made as a platform that can facilitate advanced security audits and penetration tests [67]. Several software tools, including those used in the final developed python script, come preinstalled with Kali Linux. In addition, the distribution has a number of word lists containing commonly used passwords, which were employed to crack the WiFi keys.

### 3.1. Hardware

In addition to the hardware presented by a regular laptop itself, a few additional requirements must be satisfied for the attack to succeed. Most importantly, a wireless NIC capable of being set to “monitor” mode is required. This NIC will serve as an interface from which most of the steps in the Python script will be launched. In the final step of the client takeover, another NIC is required to run the rogue AP. While this could be run on the wireless card with monitoring capabilities, this card is used to kill the connection between the original AP and the client while the rogue AP is running. The laptop used was a “Lenovo ThinkPad T430 2349-EH9” with a Intel Core i5-3320M 2.60GHz CPU, and an Intel Centrino Advanced-N 6205 Dual Band NIC. The additional external USB NIC was a Linksys AE1200.

### 3.2. Software

All software used in the Python program, including the Python libraries, which were mostly from the Python Standard library, comes preinstalled with Kali Linux. The following is an overview of the software tools used.

#### 3.2.1. The Aircrack-ng Suite

Aircrack-ng is a suite of programs written in shell code that can be used to test the security of the most common encryption methods used in Wi-Fi today. The suite is a free, open-source, terminal run set of tools that is continuously maintained and utilized. The suite consists of several tools including Airmon-ng, Airodump-ng, Aireplay-ng, Aircrack-ng, and more. The tools can be used together to perform various actions including hardware analysis, packet monitoring and injection on the wireless interface, and performing brute-force and dictionary attacks against WEP and WPA-PSK encryption keys. It can also spoof an AP, and trick a client computer into connecting to its own rogue AP imitating the target Wi-Fi. The specific tools mentioned above were those of the Aircrack suite used in this project. Below is a description of their applications in the scope of this project.

#### 3.2.2. Airmon-ng

This tool gathers and displays information about the computer network cards, interfaces, and network processes. It can change the mode of the computer wireless cards to "monitor" mode which enables them to read and inject packets on the air, regardless of their source and destination. It can also kill the running network process that may interfere with the wireless interfaces while they are used by other tools. While it is not essential to run for most of the other tools to work, it creates a more stable foundation for them to operate on.

#### 3.2.3. Airodump-ng

**Figure 3:**
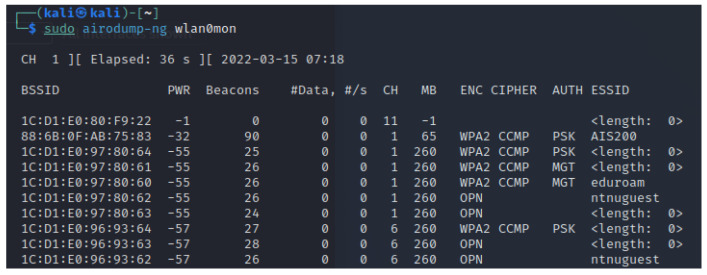
Airodump-ng running in terminal.

When wireless cards are operating in monitor mode, Airodump-ng is capable of sniffing any packets transmitted within range over the air interface. As a baseline, this can be used to get an overview over all APs and probes in the area. The information gathered on each node will include the ESSID, the BSSID, the encryption scheme, the channel on which it operates, its relative signal strength, and its connected stations. Airodump can also be configured to narrow its scope to a single AP and its clients, for instance, to capture initialization vectors or WPA handshakes. The captured data is displayed in the terminal as shown in Figure 3, or can be written to files.

#### 3.2.4. Aireplay-ng

Aireplay-ng can be used to inject packets and spoof their source. One particular useful application in the scope of our project is to transmit deauthentication packets to clients. These packets claims to be from their connected AP, resulting in the clients dropping their connection. By continuously transmitting these packets, the client will not be able to reconnect with the AP, effectively executing a DOS attack. If no further deauthentication packets are transmitted, the clients will attempt to reestablish their connection through a new authentication process. During this process, a wireless sniffer like Airodump-ng may capture the handshake.

#### 3.2.5. Aircrack-ng

**Figure 4:**
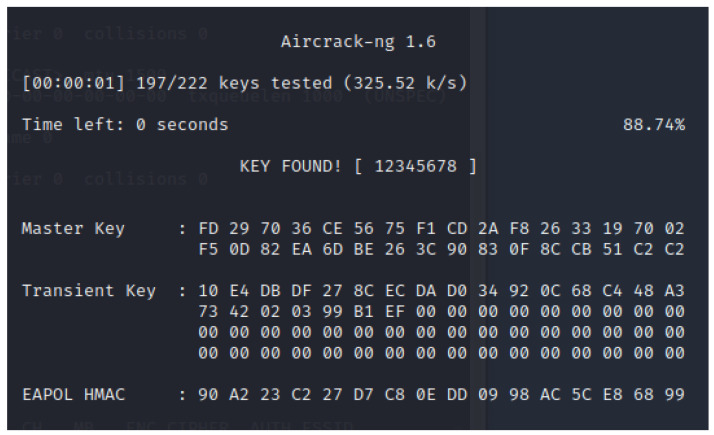
Aircrack-ng cracking a WPA2 key.

With the same name as the tool suite itself, Aircrack-ng is used to crack WEP and WPA-PSK encryption keys. To retrieve WEP keys, several methods can be used. In terms of cracking a WPA-PSK key, Aircrack can use information captured from the four-way handshake, along with the BSSID of the AP. With this data, the tool performs a dictionary attack, testing several thousand keys each second, and outputs its result in the terminal or to a file. When a WPA2 is cracked, the output will look similar to the screenshot shown in Figure 4.

#### 3.2.6. Hostapd

Hostapd, or “Host Access Point Daemon”, enables communication between various 802.11 wireless access points when operating in Host AP mode [68]. It allows us to run our own AP from a wireless NIC with the name, encryption scheme, and password that we specify, in addition to numerous other parameters Airbase-ng may seem to be a sufficient tool for this purpose. Unfortunately, it is unable to host a functional WPA2 encrypted AP.. The daemon reads its configuration from a text file specified upon launch. This tool allows us to create a rogue AP that resembles the target AP enough that the client will unknowingly reconnect to it if the original connection is temporarily lost.

### 3.3. Target IoT Device

**Figure 5:**
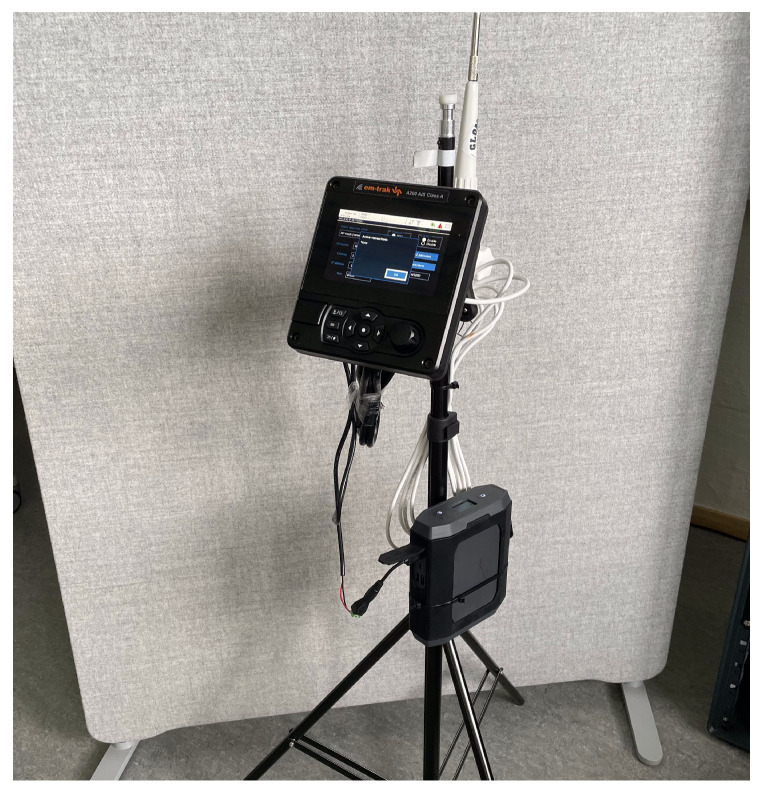
The A200 connected to a battery and a VHF antenna.

The IoT device used in the project is the AIS displayed in Figure 5. It is the A200 AIS Class A produced by Em-Trak [69]. An AIS is a autonomous monitoring and tracking system that communicates positional data and vessel information with nearby harbors and ships [70]. The AIS makes an operator able to see the location of other vessels in vicinity, aiding with navigation and collision avoidance. It is used on larger ships and utilities ashore like VTS for tracking, monitoring, and identification. The AIS transmits data continuously and is required by international treaties to be installed and operational for larger vessels.

**Figure 6:**
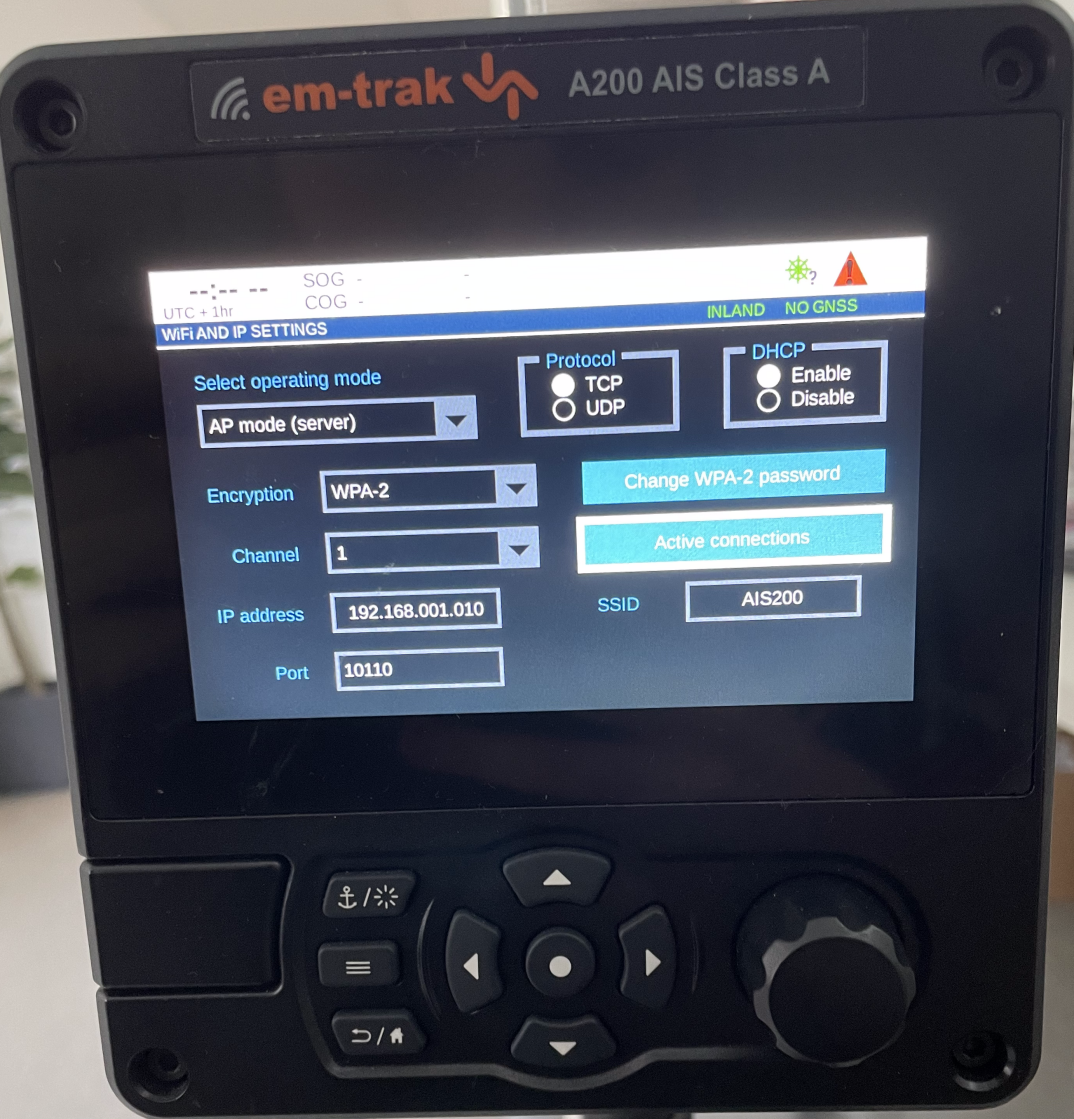
The Wi-Fi settings menu in A200.

When mounted on a ship, the A200 AIS is connected to several sensors on the ship along with a VHF antenna for communication with other AIS systems. It has a wireless NIC which it uses to run its own Wi-Fi AP. The Wi-Fi configuration menu is displayed in Figure 6. From there, the user can set the ESSID, choose internet protocol, select channel, and more. A ship operator can access and read data from the device by connecting to it with a laptop, tablet or similar device. The data is transmitted using NMEA 0183 messages which are continuously sent to all clients on the network. To read the NMEA messages, the client needs some kind of chart plotter software. There are many alternatives, but OpenCPN was used for this project [71]. The connection is protected with WPA2 Personal by default, which is the highest level of encryption the device supports. There is no password complexity validation.

The Em-Trak A200 AIS Class A is an expensive AIS device used for larger vessels like freight and passenger ships and is priced at almost £2000 [69]. The tested device is the current version in production and market, shipped with a three year global warranty. Thus, we can expect this model to be operative in its current state in many years to come.

### 3.4. Physical Setup

In the facilities of the NCR we set up the testing environment. The A200 device was enabled in Wi-Fi mode with an Apple MacBook running the operating system MacOS Monterey as the client. They were located one meter apart from each other, with no physical objects between them. The laptop with Kali Linux was placed about one meter away from both the A200 and the MacBook. The AP options were left as displayed in Figure 6, which are the default values.

**Figure 7:**
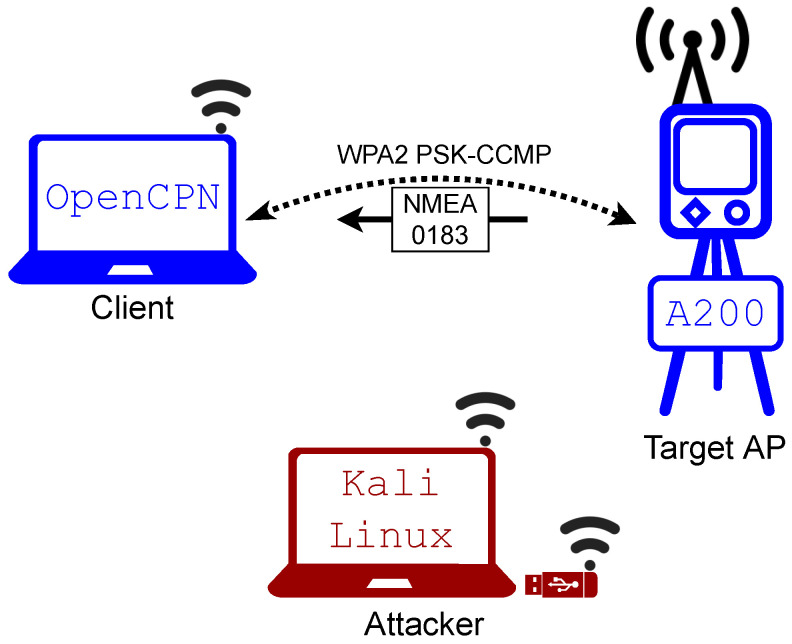
Diagram of the case study setup.

**Figure 8:**
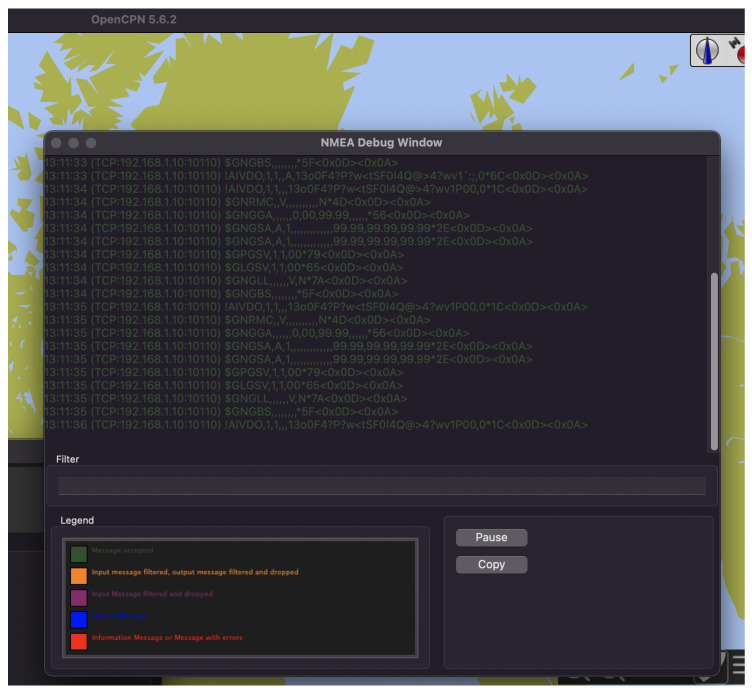
OpenCPN debug window with received NMEA packets.

Because the AIS was not connected to all of its sensors, the messages that it transmitted had no geographical data that could be displayed in the chart plotter. However, the debug window within OpenCPN displays all received NMEA messages, which was sufficient to prove the wireless connection and possible interruptions. Figure 7 displays a diagram of the case study setup, while Figure 8 shows the debug window of OpenCPN while receiving packets over a Wi-Fi connection.

## 4. Manual Attack Results

**Figure 9:**
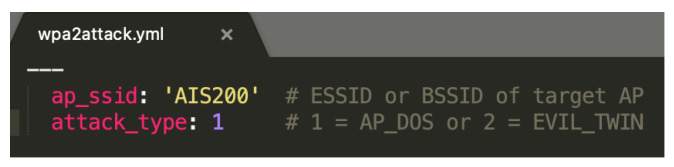
YAML file for a DoS attack.

To launch an attack, the agent must know the ESSID or BSSID of the target AP. This information is provided using a YAML file. Figure 9 displays the contents of a YAML file for launching a DOS attack on a network named “AIS200”. Below are the results of each attack.

### 4.1. Attack 1: DoS

**Figure 10:**
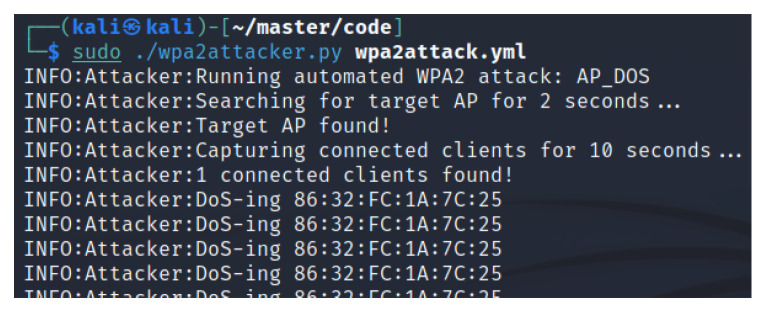
Performing the DoS attack.

**Figure 11:**
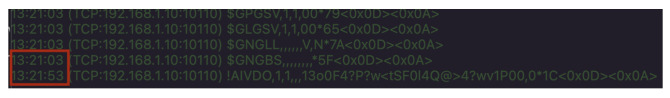
DoS attack in OpenCPN debug window.

The MacBook was connected to the A200 AP as described in Section 3.4, continuously receiving NMEA 0183 packets every second which were interpreted using OpenCPN. The ESSID of the network was “AIS200”, which is the default setting, and the client had the BSSID “86:32:FC:1A:7C:25”. The DOS attack was launched and sustained for 45 s, with the terminal output displayed in Figure 10. The agent used 18 s to gather the information required to launch the attack, resulting in a complete program run time of 1 min and 3 s. In Figure 11, we can see a sudden time gap between received NMEA packets of 53 s. The additional 8 s comes from the time it took for the client to reestablish its connection.

### 4.2. Attack 2: Evil Twin

**Figure 12:**
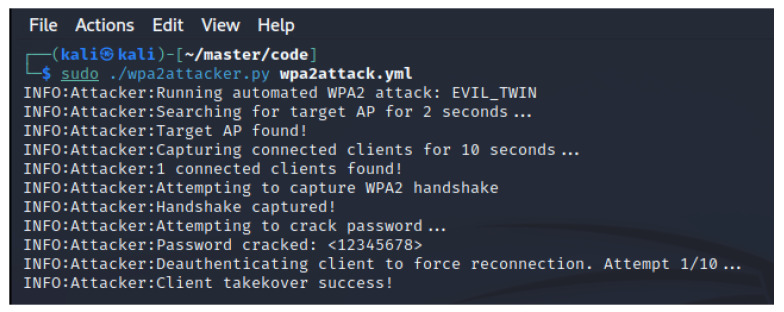
Performing the Evil Twin attack.

**Figure 13:**
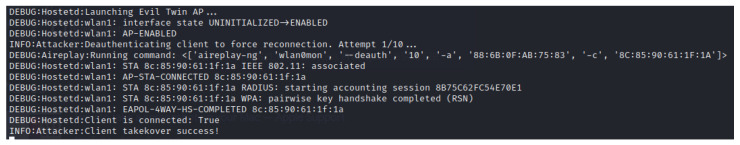
Debug view of Evil Twin connection.

Again, we followed the setup previously described. The network ESSID remained unchanged, but the client BSSID was “8C:85:90:61:1F:1A” for this experiment. “12345678” was used as the network encryption password. As seen in Figure 12, the agent was able to successfully impersonate the A200 AP and trick the client into connecting to it. The reconnection was done automatically and transparently by the client MacBook operating system without interaction from the user. When running the attack in verbose mode, we can see the client connection in the debug logging messages as displayed in Figure 13. The command for deauthenticating the client using Aireplay-ng is also visible in the screenshot. The run time from start to client connection takeover was 39 s. A notable variable considering the result is the time it took to crack the password. Aircrack-ng used less than one second to find “12345678” in the provided wordlist. This due to the size of the wordlist, which contained less than 200 words. A discussion of this matter is found in Section 7.

## 5. System Design for Automated Attacks

Two similar attacks on the A200 IoT device were designed and implemented. Both are based on vulnerabilities in the WPA2 encryption scheme, targeting the network layer of the device. We have chosen these attacks because they affect all implementations of Wi-Fi using WPA2, and can be performed without extensive knowledge, expertise, or equipment. This Section will describe the attacks, define their EP models, and verify them using TLA+ and the TLC Model Checker. Finally, the verified design is implemented in Python.

### 5.1. The Attack Procedure

**Figure 14:**
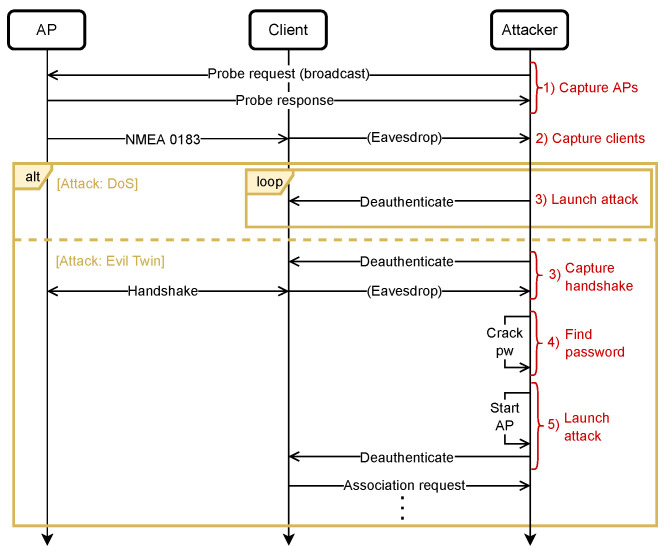
Sequence diagram of DOS and Evil Twin attack.

The first attack is a DOS attack using deauthentication frames, and the second is an Evil Twin attack. Figure 14 displays a sequence diagram of the two attacks. As evident from the diagram, the first two steps in both procedures are equal.

**Attack 1: DOS**
  1. **Capture APs**
    First, the agent will capture all APs within range. This can be done within a couple of seconds using Airodump-ng but should be performed on a monitoring network interface. To change the mode of NIC, the agent will use Airmon-ng.
  2. **Capture clients**
    If the target AP is found in previous step, the agent will capture the clients connected to the AP using Airodump-ng. Depending on the traffic on the network, this may take few more seconds than capturing the APs.
  3. **Launch attack**
    Provided that there are clients on the network, the agent should perform the DOS attack. Each client will continuously receive deauthentication frames that appear to be from the AP, withholding them from the reconnecting.
**Attack 2: Evil Twin**
  1. **Capture handshake**
    This step involves capturing the handshake process between a client and the AP. By deauthenticating the client using Aireplay-ng, the agent can capture the handshake upon reconnection using Airodump-ng.
  2. **Find password**
    The password can be cracked using a dictionary attack if the nonces of the handshake was captured in the previous step. To perform the dictionary attack, Aircrack-ng will be used.
  3. **Launch attack**
    If the password is cracked, the agent will spoof the network of the target AP using Hostapd. When deauthenticating the client again using Aireplay-ng, the client should automatically reconnect to the Evil Twin AP if its signal strength is stronger than that of the true AP.

### 5.2. EP Models of Attacks

**Figure 15:**
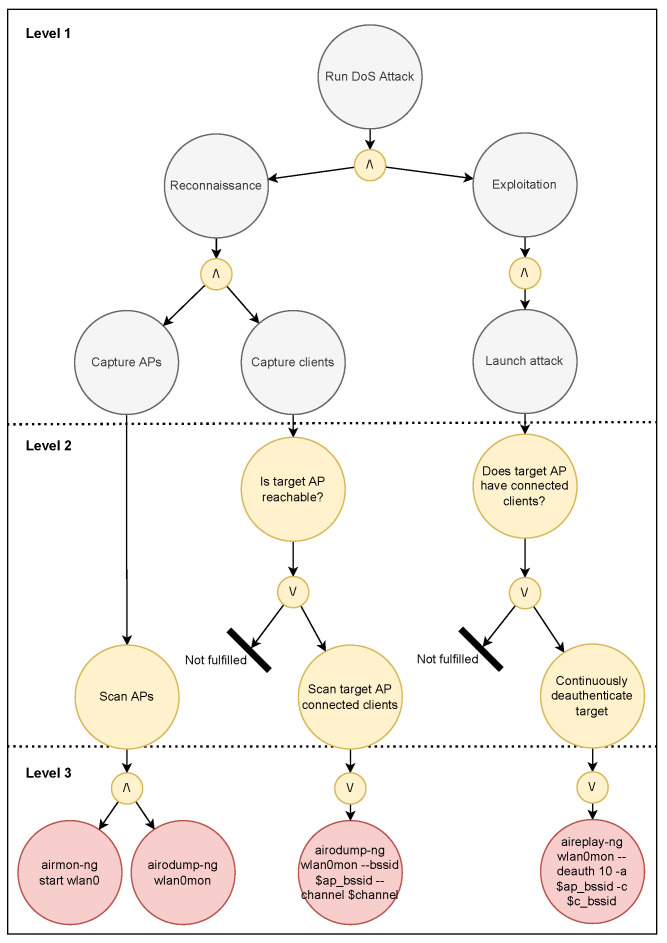
EP model of the DOS attack.

**Figure 16:**
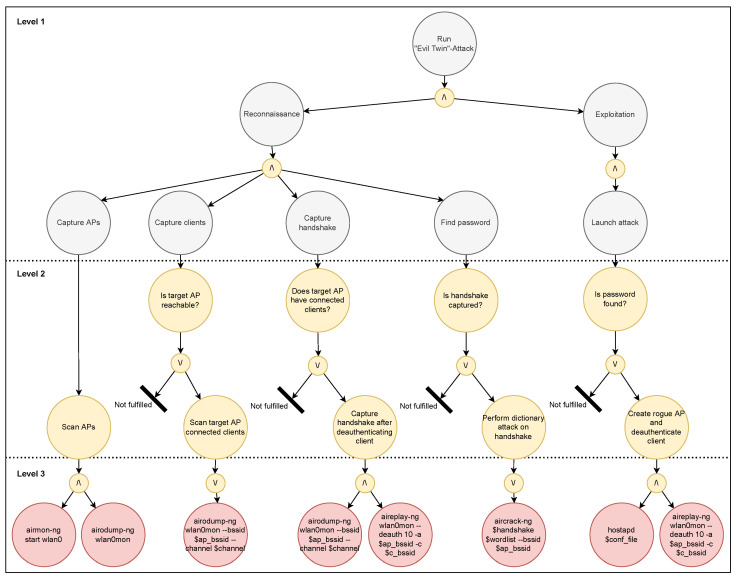
EP model of the Evil Twin attack.

The attack was performed through an agent that was designed based on the EP modeling discussed in Section 2.4. The diagrams displaying the high level abstraction of the DOS and Evil Twin attack models are shown in Figure 15 and Figure 16, respectively. When running the attack, the agent should analyze the main goal, which is either Evil Twin or DOS, and then attempt to fulfill the subgoals subsequently read from left to right in the model. Because both attacks involve sequential tasks where either a plan is fulfilled or the program terminates, this specific EP model is relatively simple with a single condition in the second level of each subgoal.

## 6. System Implementation and Formal Verification

In the work of Yamin and Katt, Datalog [2] was used for formal modeling and verification of the EP. In this work, TLA+ were chosen as the formal language because it presents an approach that is based on theoretical mathematics and is thus more expressive. While Datalog is a language mainly used for database querying capable of verifying models, TLA+ was designed for modeling hardware and software at an abstract level [45,72]. Furthermore, a software called *TLA+ Toolbox* provides both the *TLC Model Checker* which can verify the formal model, as well the possibility to create state transition diagram of the results.

**Figure 17:**
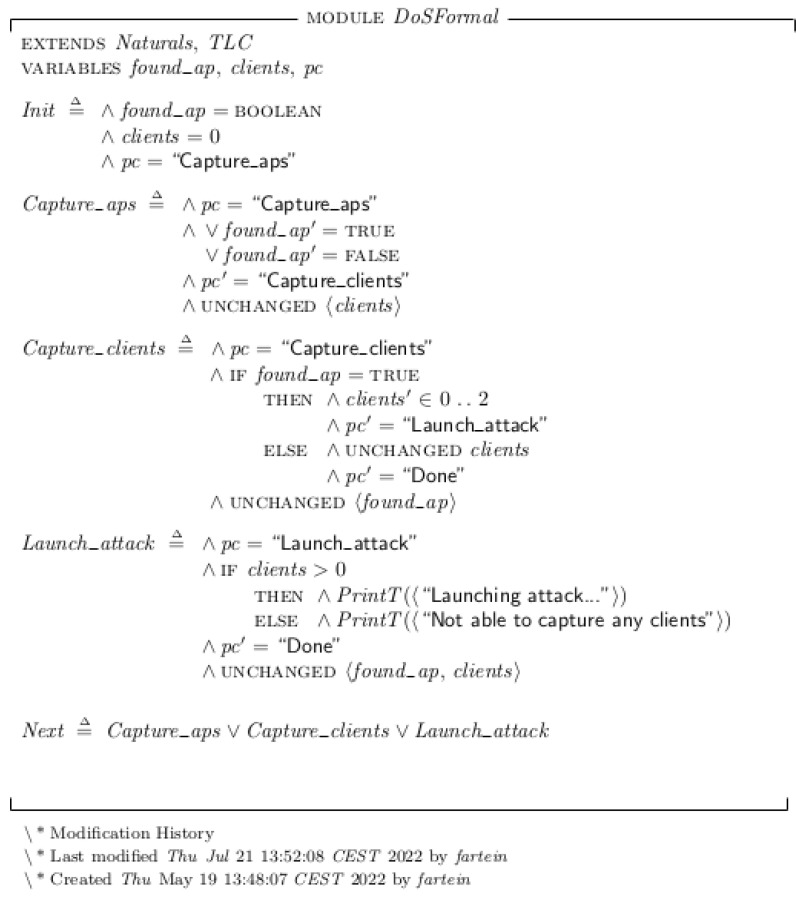
The formal specification of the DOS attack EP model written in TLA+.

**Figure 18:**
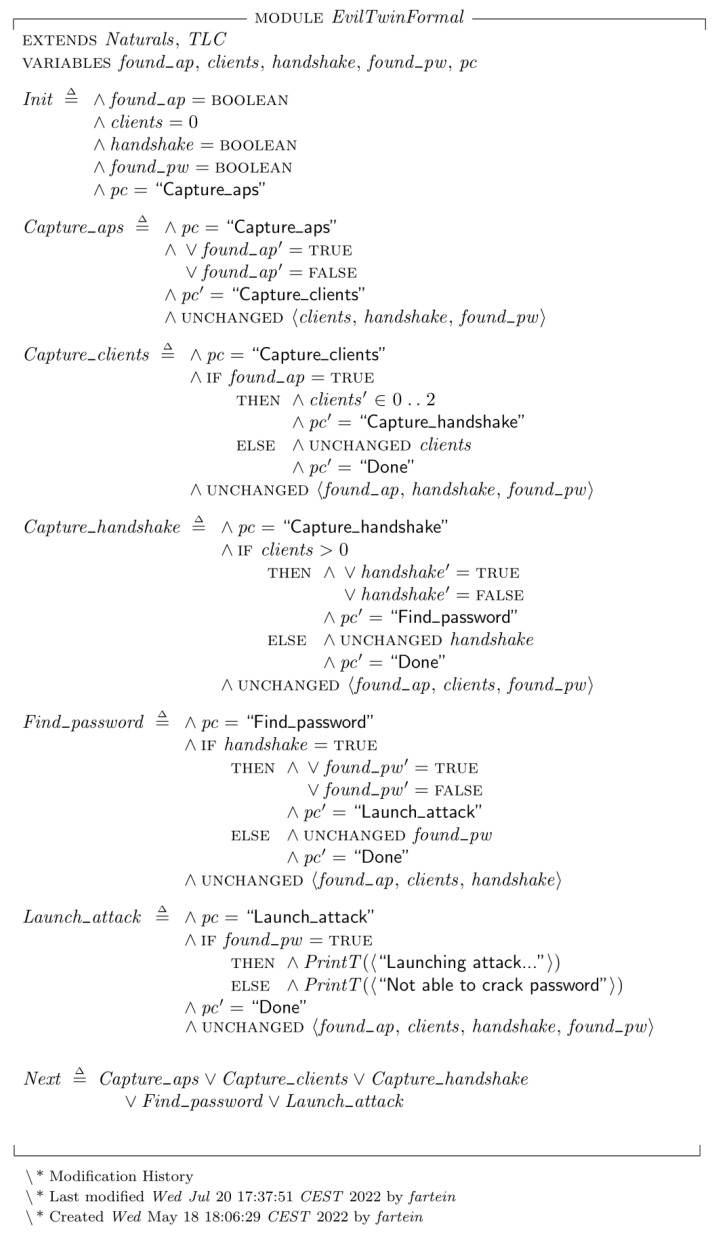
The formal specification of the Evil Twin attack EP model written in TLA+.

The model complexity for a single Evil Twin attack in this setup is trivial to the point where the formal model verification is practically unnecessary. However, its value becomes evident in complex systems where one or more agents are capable of performing many different attacks and intelligently choosing between them based on the reconnaissance phase. The formal models of the attacks written in TLA+ are presented in Figure 17 and Figure 18. Each next-state relation definition within the specification translates to a subgoal branch of the EP model. The actions represented by the bottom node within the second layer of the model defines the variable changes of the state. For instance, the “Capture clients” subgoal node in Figure 16 is described by the next-state relation “Capture_clients” in Figure 18. Within this relation definition, the condition “Is target AP reachable” of the EP model is tested by the operation “IFfound_ap=TRUE”. If this is found true, the action “Scan target AP connected clients” is performed by giving the variable “clients” an integer from 0 to 2, indicating the number of captured connected clients The max limit of clients should in theory be equal to the maximum number of possible clients connected to the AP but was set to 2 because it would limit the state diagram to where it was possible to display within the thesis. During testing, we also set the limit to 100 devices to ensure that the validity of the model did not change.

The level three commands of the EP model is not described by the specification, but by the abstraction defined in the action node of the level above. This corresponds to the TLA+ concept of abstracting the functionality and conforms to explaining *what*s of the system, leaving the *how*s to the lower level code implementation. In the final state of an EP where the last plan and subgoal was fulfilled, the specification prints “Launching attack…” to illustrate a successful run. In the cases where the plan was successful until the very last state but failed the last condition, the specification prints either “Not able to crack password” or “Not able to capture any clients” to indicate that the plan was not fulfilled.

**Figure 19:**
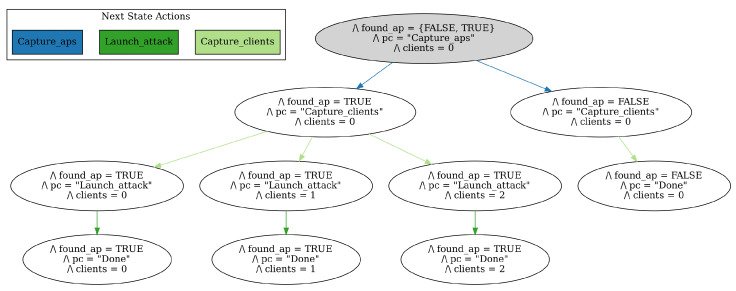
State transition diagram showing all possible states of the DOS attack EP model.

**Figure 20:**
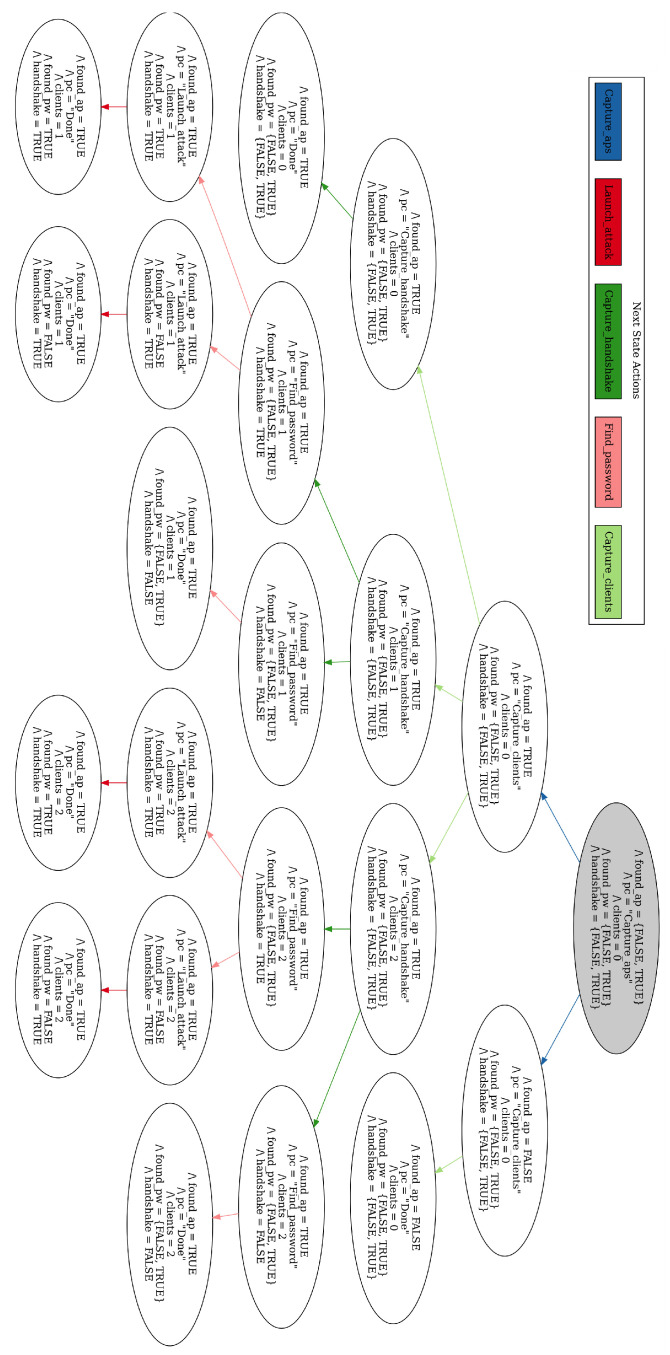
State transition diagram showing all possible states of the Evil Twin attack EP model.

First, we wanted to formally verify that the logic of the TLA+ specifications, and consequently, the EP models and agent decision making processes, were not flawed. This includes situations were the program terminates without reached a “Done” state, or where it enters an infinite loop. Second, we wanted to verify that the final states of the models could be theoretically reached. For verification, we used the TLC Model Checker. The model checker was able to compile and run the specifications without errors, which ensures there are no logical flaw in the specifications. We could see from both the printed output of the model and generated state transition diagrams that the final states were reachable. The state transition diagrams for the DOS and Evil Twin attacks are displayed in Figure 19 and Figure 20, respectively. When running model checker for the DOS attack, the following output was produced:

<<“Launching attack…”>>
<<“Not able to capture any clients”>>
<<“Launching attack…”>>

These strings represent the final three possible states seen in the corresponding state diagram where the agent found the target AP. There are three of them because there are three possible values for the number of clients found in the previous state. The states in which the model found one or two clients were able to launch the attack while the third did not. Thus, the specifications and EP models were verified.

### 6.1. Implementation

**Figure 21:**
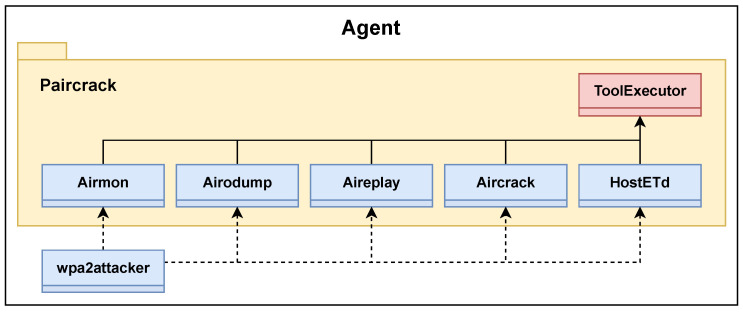
Class diagram of the agent.

The agent was implemented in Python 3.10. An essential part of the program is the *Subprocess* module from the Python Standard library [73]. The module was used to create and communicate with the processes running the software tools presented in Section 3.2. To ensure modifiability and applicability in other implementations, the program was written with high modularity following an object oriented approach. Figure 21 shows a simple class diagram of the program.

#### 6.1.1. Tool Interface

A custom package named *Paircrack* was created and used as an interface between the agent decision making process and the software tools used. Each class within the package corresponds to a tool from the Aircrack-Ng suite or HostAPd. Because of common functionality between the package classes, an abstract class named *ToolExecutor* was created. Most importantly, this class runs and interprets the outputs of the individual tool processes. In Algorithm 1, the function for scanning and capturing APs is presented. This function uses the helper function “_capture” which calls the “run” function of the ToolExecutor class. All classes in the Paircrack package call the “run” function to start its processes. A part of this function which uses the Subprocess module is displayed in Algorithm 2. Because some processes run until they are manually interrupted, like those started by Airodump-Ng, a process timeout ensures their eventual termination.

A Python package for running Aircrack-Ng programs named  *Pyrcrack* [74] already exists, and it serves many of the purposes we have implemented in Paircrack. While originally intended to be used as the tool interface and foundation for the agent, the package was discarded due to limited documentation and difficulties of use. Pyrcrack is still under development, which may make it applicable in future generations of the agent. The custom Paircrack package used in this project has some features inspired by Pyrcrack.

**Algorithm 1.** The function for capturing all APs within range.
def capture_aps(self, interface : str, proc_timeout=2) -> str:

    “““Captures all access points within range
 
    Parameters

    ----------

    interface : str

        Interface to capture packets on

    proc_timeout : int, optional

        Amount of seconds to run the capture, by default 2
 
    Returns

    -------

    str

        filepath to xml file containing AP data

    “““
 
    self.logger.debug(’Capturing all APs...’)

    fsuffix = []

    flags = {

        ’--write-interval’: ’1’,

        ’--output-format’: ’netxml’}

    return self._capture(interface, fsuffix, flags, proc_timeout)


**Algorithm 2.** Part of the “run” function within the abstract ToolExecutor class.
self.logger.debug(f’Running command: <{command}>’)

if self.verbose:

    self.logger.debug(f’\tkeywords: <{proc_flags}>’)
 
try:

    output = subprocess.run(command, **proc_flags)

except subprocess.TimeoutExpired as e:

    self.logger.debug(f’Process timout’)

    return True

else:

    if self.verbose:

        self.logger.debug(f’Captured stdout: <{output.stdout[:-1]}>’)

        self.logger.debug(f’Captured stderr: <{output.stderr}>’)

    return output


#### 6.1.2. Agent Decision Making

The agent decision making process discussed in Section 5.2 was implemented in the class *wpa2attacker*. This class creates instances of the classes within the Paircrack package, and use their functionality to execute the actions described in the EP model. To determine what type of attack to run, i.e., DOS or Evil Twin, the attack type is given as program input in a YAML file. This file also contains a string that can be either the BSSID or the ESSID of the target AP. The YAML file is the only input required for the agent to run either attack. Providing the initial configuration data in a YAML file is, for compatibility reasons, conducted with the framework and setup first created in the work of Yamin and Katt by matching the interface with that of the autonomous agents on which this work is based.

## 7. Discussion

The case study was performed in a controlled environment. Because of this, we will address some important factors the could change the results of the Evil Twin attack if performed in a realistic scenario.

### 7.1. Network Interface Range

A potential limitation of the attack efficiency comes from the distance between attacker, client, and AP. The client will only connect to the rogue AP if the AP signal strength is stronger than that of the authentic AP. If the signal strength of the impersonated network is weaker, the client will simply reconnect its original AP. Unless the attacking device is onboard the ship, it is likely to be further away from the client than the AIS is. On the other hand, this issue can be addressed by either jamming the authentic AP [75], or increasing the strength of the wireless signal [76]. In this work, an old and simple network adapter was used to host the rogue AP. Even in its default signal strength configuration, the client is connected to it. With a new and better adapter, the wireless signal strength would be stronger, which could be further increased by modifying the signal strength.

### 7.2. Time to Crack Network Password

**Figure 22:**
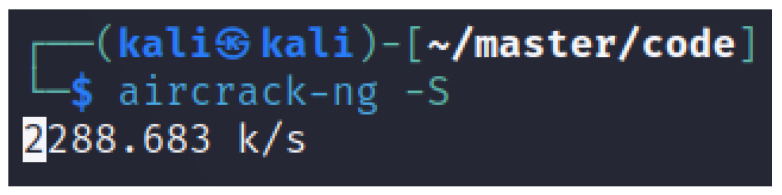
Aircrack-ng password cracking speed on Kali Linux machine.

**Figure 23:**
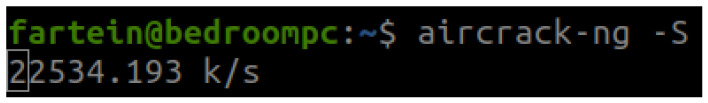
Aircrack-ng password cracking speed on the desktop computer.

In the case study, we used a wordlist with 200 words where we knew the AP (Access Point) password was one of them. Because of this, the time it took to crack the password was less than one second, which would certainly not be the case in a real scenario. The number of keys tested per second on the case study computer using Aircrack-ng amounts to approximately 2300. Testing the cracking speed on desktop computer with an AMD Ryzen 5 3600 6-Core processor, we saw an increase to about 22,500 keys per second. However, these numbers could be outperformed by software tool for hash cracking called Hashcat using GPU acceleration. According to a speed test on Hashcat using GPU services provided by Google Cloud, the tool can test 1.1 million WPA2 keys per second when running on Nvidia-Tesla-a100 [77]. Using these password cracking speeds to calculate the time it takes to exhaust different wordlists, we get the results as presented in Table 1. By using the Aircrack-ng on the Kali Linux machine, a wordlist with the same size as the famous “rockyou.txt” can be exhausted within minutes. Even a random eight integer password is feasible to crack. However, Aircrack-ng would not be able to crack a random password of integers and letters of eight characters. By using Hashcat, attacks on wordlists with millions of passwords or even random letter passwords would be possible as indicated in Figure 22 and Figure 23.

### 7.3. Comparison with Similar Systems

In 2018, researchers [16] proposed the idea of automated penetration testing of IoT devices. They discussed the general threats faced by IoT devices and ran a simulation to validate those threats. There initial results were useful; however, they required experimental validation in real IoT environment. In 2020, researchers [25] performed a study in which they performed penetration testing on real IoT devices. They proposed a general methodology to develop test cases for penetration testing IoT devices and planned to automate the test cases in future studies. Similarly, in a recent 2022 study [78], researchers proposed an expert system that takes IoT infrastructure details as an input and suggest a threat model and a penetration testing plan. A penetration tester then can utilize the plan to systematically test the IoT infrastructure. Comparing to the above mentioned work in our study we first formally model and verify different IoT threats and then automatically execute the attack on IoT devices with minimal human involvement.

## 8. Conclusions

In this work, we investigated vulnerabilities in IoT devices. We developed an autonomous agent whose decision-making process was based on the EP model. The agent decisions models were verified using the formal language TLA+. By successfully launching the two attacks in a case study involving a currently employed IoT device, we have demonstrated that the EP model can be used to automate penetration testing. The results indicated that the agents were not only able to put the target device out of service but also spoof the connection to the client. Only considering the fact that we could easily and automatically kill all wireless connections to the device proves its security to be inadequate. As mentioned, the device is used on large vessels like yachts, cruise ships, and freight ships, and international maritime law requires it to be operative at all times. Hourly docking fees, labor, and maintenance costs are high for these kinds of ships. If the navigational device is put out of service for only a few hours, the associated extra costs can amount to thousands of dollars, not to mention that many of these ships are considered critical infrastructure. The particular device tested is a high end, off-the-shelf product with three year warranty, and may be employed for many more years as discussed in Section 2.1.3.

Furthermore, by being able to make the client automatically and transparently connect to a rogue AP, the issue increases in severity. In the case of AIS devices, sending fake GPS data can lead to collisions with harbors, underwater reefs, or other ships. The connection can also be misused to send malware or in other ways attack the client. In the case of routers or other devices providing Internet access, the connection can be used for MITM attacks. As IoT devices are used in anything from private homes to critical infrastructure, the fact that many of them are using insecure methods of communication and may continue to do so for several years to come should be an alarming conclusion. As we have discussed and seen, WPA2 is inherently vulnerable. To mitigate the vulnerabilities presented in this work, Wi-Fi networks should utilize the more secure WPA3. If WPA2 must be used for compatibility or other reasons, strong password policies should be employed. As displayed in Table 1, if the password length is sufficiently long and hard to guess, it is infeasible to crack, even with cloud provided GPU accelerated hash cracking tools.

As attackers develop new methods to compromise systems, automated penetration testing tools will need to evolve to identify and exploit these new vulnerabilities. At the same time, automated testing tools will need to be updated to identify and defend against new attack methods. Automated penetration testing tools may be integrated with other security tools and processes, such as vulnerability management, incident response, and compliance management. Automated tools may use machine learning and artificial intelligence to improve their ability to identify and exploit vulnerabilities, leading to greater integration with other security tools and processes.

## Figures and Tables

**Table 1:** Comparison of key test speeds on different wordlists using Aircrack-ng and Hashcat.

	Aircrack-ng, Kali Machine	Aircrack-ng, Desktop	Hashcat
**Crack speed (k/s)**	2300	22,500	1.1 million
**Dictionary attack on** **1.4M key wordlist (s)**	10 m 6 s	62 s	1 s
**Brute-force 8 integers**	13 h	75 min	2 min
**Brute force 8** **lowercase letters**	3 years	4 months	53 h
**Brute force 8 integers** **and lowercase letters**	40 years	5 years	30 days
**Brute force 12 integers** **and lowercase letters**	66,234,755 years	6,770,664 years	138,491 years

## Data Availability

Not applicable.

## Abbreviations

The following abbreviations are used in this manuscript:

AES	Advanced Encryption Standard
AI	Artificial Intelligence
AIS	Automatic Identification System
AP	Access Point
APT	Advanced Persistent Threat
BDI	Belief–Desire–Intention
BSSID	Basic SSID
CCMP	Counter Mode with Cipher Block Chaining Message Authentication Code Protocol
DDoS	Distributed Denial of Service
DoS	Denial of Service
DSR	Design Science Research
EAPOL	Extensible Authentication Protocol over LAN
EP	Execution Plan
ESSID	Extended SSID
GPS	Global Positioning System
GTK	Group Temporal Key
IDS	Intrusion Detection Systems
IEEE	Institute of Electrical and Electronics Engineers
IGTK	Integrity Group Temporal Key
IIoT	Industrial Internet of Things
IoMT	Internet of Medical Things
IoT	Internet of Things
IPS	Intrusion Protection Systems
LAN	Local Area Network
MIC	Message Integrity Code
MitM	Man-in-the-Middle
NCR	Norwegian Cyber Range
NIC	Network Interface Card
NMEA	National Marine Electronics Association
PMK	Pairwise Master Key
PSK	Preshared Key
PTES	Penetration Testing Execution Standard
PTK	Pairwise Transient Key
RL	Reinforcement Learning
SSID	Service Set Identifier
VHF	Very High Frequency
VTS	Vessel Traffic Services
WEP	Wired Equivalent Privacy
WLAN	Wireless LAN
WPA	Wi-Fi Protected Access
